# Evolution of the SpoIISABC Toxin-Antitoxin-Antitoxin System in Bacilli

**DOI:** 10.3390/toxins8060180

**Published:** 2016-06-09

**Authors:** Marek Gabriško, Imrich Barák

**Affiliations:** Institute of Molecular Biology, Slovak Academy of Sciences, 845 51 Bratislava 45, Slovakia; marek.gabrisko@savba.sk

**Keywords:** *Bacillus subtilis*, toxin-antitoxin, *Bacillus cereus*, SpoIISABC, molecular evolution

## Abstract

Programmed cell death in bacteria is generally associated with two-component toxin-antitoxin systems. The SpoIISABC system, originally identified in *Bacillus subtilis*, consists of three components: a SpoIISA toxin and the SpoIISB and SpoIISC antitoxins. SpoIISA is a membrane-bound protein, while SpoIISB and SpoIISC are small cytosolic antitoxins, which are able to bind SpoIISA and neutralize its toxicity. In the presented bioinformatics analysis, a taxonomic distribution of the genes of the SpoIISABC system is investigated; their conserved regions and residues are identified; and their phylogenetic relationships are inferred. The SpoIISABC system is part of the core genome in members of the Bacillus genus of the Firmicutes phylum. Its presence in some non-bacillus species is likely the result of horizontal gene transfer. The SpoIISB and SpoIISC antitoxins originated by gene duplications, which occurred independently in the *B. subtilis* and *B. cereus* lineages. In the *B. cereus* lineage, the SpoIIS module is present in two different architectures.

## 1. Introduction

Programmed cell death (PCD) is a genetically-regulated system in which a bacterial cell is able to commit suicide in response to a variety of different stresses. This response includes cell lysis or growth inhibition induced by harsh environmental conditions, such as starvation or antibiotic treatment, active mother cell lysis during sporulation to release the spore or altruistic suicide to release cell content to provide the nutrients required for the normal development of the remaining bacterial population [1]. PCD is usually mediated by a toxin/antitoxin (TA) genetic pair. However, there is controversy about the role of TA systems in PCD, and it was proposed that these systems more likely enhance the formation of dormant persister cells [2]. Toxins are always highly stabile proteins. Their antidotes, the antitoxins, are usually labile proteins or small RNAs. TA systems are classified according to the nature of the antitoxin. Antitoxin types I and III are small RNAs, which either inhibit toxin synthesis or capture it. Types II, IV, V and VI, on the other hand, are all proteins that are distinguished based on their mode of action. The type II antitoxins are in many cases small proteins with an *N*-terminal DNA-binding domain and a *C*-terminal toxin-bonding domain; the type IV antitoxin is an antagonist of its cognate toxin and competes with it in binding to its target; the type V antitoxin is an endoribonuclease that degrades the toxin-encoding mRNA [3]; and the type VI antitoxin acts as an adaptor by binding to its toxin partner and induces its proteolytic degradation [4].

The SpoIIS system was discovered during a study of *Bacillus subtilis* cell differentiation and originally appeared to consist of two proteins, the SpoIISA toxin and the SpoIISB antitoxin [5]. SpoIISA is a 248 residue-long protein with three putative transmembrane segments at its *N*-terminus and a cytosolic domain at its *C*-terminus. Sporulating *B. subtilis* cells synthesizing SpoIISA in the absence of SpoIISB exhibit lethal damage to the sporulation septum; the cytoplasmic membrane of vegetatively growing *B. subtilis* cells is also susceptible to the toxicity of overexpressed SpoIISA [5,6]. Interestingly, the SpoIISA is not toxic to only Gram-positive bacteria. The SpoIISA protein from *B. subtilis* and its homologues from *B. cereus* and *B. anthracis* are also capable of killing Gram-negative, two-membrane *Escherichia coli* cells. This phenotype can be prevented by the formation of a tight complex between the cytosolic domain of the toxin and its small, hydrophilic SpoIISB antidote [5,7]. SpoIISB is a small, 65 residue-long protein with no obvious tertiary structure [7]. The toxin-inhibitor complex adopts a heterotetrameric configuration, with two SpoIISB inhibitors wrapped around a SpoIISA dimer. The entire SpoIISB sequence is involved in extensive interactions with SpoIISA, and each SpoIISB protein interacts with both units of the SpoIISA dimer [7]. Recently, a third component of this system, SpoIISC, was identified, possessing several properties similar to SpoIISB. Both are small proteins and both are capable of neutralizing the toxicity of SpoIISA and, thus, serve as antitoxins [8]. The genes coding for the SpoIISA, SpoIISB and SpoIISC proteins are positioned next to each other on the chromosome, likely forming a single operon. It is thought that both proteins are transcribed together from a common promoter, but SpoIISB is likely to also be transcribed from its own promoter. Because SpoIISB is probably less stable than SpoIISA; two promoters are needed to ensure that it is produced at a high enough level to compensate for its higher rate of degradation [5]. There is presently no evidence for an additional regulatory region for the transcription of *spoIISC*. To date, SpoIISA cell death-inducing activity has been observed only when both SpoIISB and SpoIISC proteins were absent or when SpoIISA was overexpressed. Since the activation of this TA system has never been observed under physiological conditions, the role of this system in programmed cell death remains unknown.

Interestingly, this TA system is widely distributed among Bacilli species. Here, we identified homologues of all *spoIIS* genes on the chromosomes of a wide range of Bacilli species, and we analyzed their possible evolution and genetic transfer. Although the SpoIISB and SpoIISC antitoxins appear to share the same evolutionary origin, they may differ in their functions and biochemical properties.

## 2. Results and Discussion

### 2.1. Evolution of the SpoIISA Toxin in Bacilli

**Figure 1:**
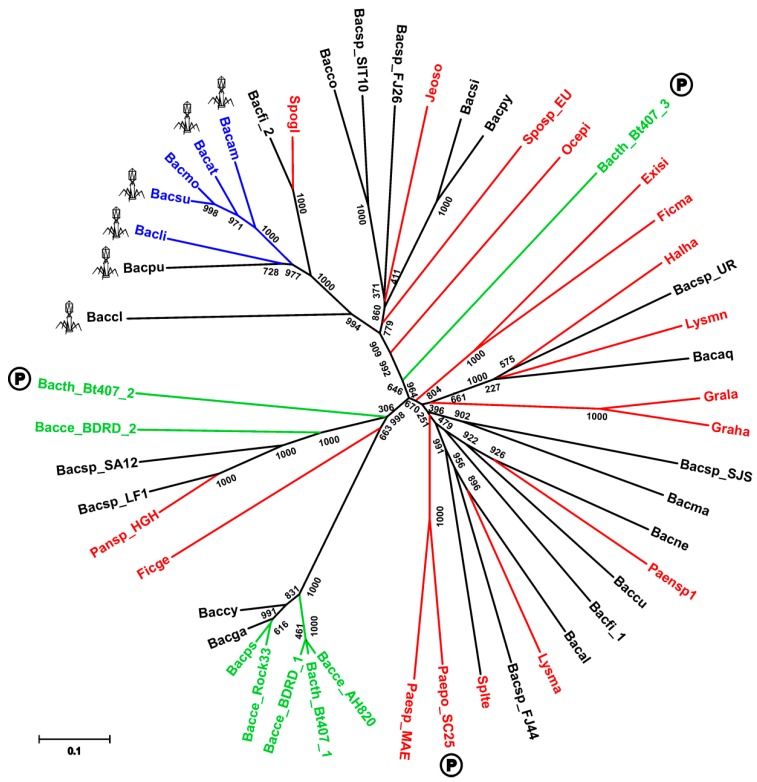
Phylogeny of the SpoIISA proteins. Neighbor-joining phylogenetic tree based on SpoIISA protein sequences from selected species. Sequences from non-bacillus species are red; those from the *B. cereus* group are green; and those from the *B. subtilis* group are blue. The abbreviations are explained in Table S2. The location of a particular SpoIISA coding gene on a plasmid is indicated by a circled P and near a PBSX prophage by a phage icon. Numbers are the bootstrap support for a particular node. The scale bar shows the evolutionary distance in the number of amino acid substitutions per site.

We found the presence of SpoIISA proteins to be restricted mostly to the Bacillus genus of the Firmicutes phylum (Firmicutes; Bacilli; Bacillales; Bacillaceae, Bacillus) (Table S1). It is notable that there are many Bacillus species whose genomes appear to lack a SpoIISA homologue (Table S1). Most of these are from unfinished genome projects, however (the genome assembly is presently in a form of individual contigs), and it is thus likely that SpoIISA proteins will be found in these genomes in the future. There are still a few species that are almost certainly missing SpoIISA. *Bacillus megaterium* is one example: the complete genomes of multiple strains (*B. megaterium* DSM 319; QM B1551; WSH-002) of this species (including 10 plasmids) are available [9,10], and none of them possess a SpoIISA protein (Table S1). Either *B. megaterium* inherited a SpoIISA protein from the common ancestor of the other SpoIISA-bearing species and subsequently lost it or this species split off from a bacilli subgroup before SpoIISA was obtained. The genomes of the alkaliphilic *B. pseudofirmus* OF4 (one chromosome and two plasmids), *B. selenitireducens* MLS10 (one chromosome) and *B. halodurans* C-125 (one chromosome) all seem to be missing SpoIISA, as well [11,12,13]. It is notable that *B. selenitireducens* does not form spores. Interestingly, SpoIISA also appears in some species outside of the Bacillus genus and some even appear outside of the Bacillaceae family (Table S1). The SpoIISA sequences from these non-bacillus species are quite dissimilar from each other, however, even if they come from closely related species (e.g., *Lysinibacillus manganicus* and *Lysinibacillus massiliensis*), which is reflected by their failure to group together in the SpoIISA phylogenetic tree (Figure 1).

Moreover, most of them are part of an accessory genome. For example, SpoIISA from *Paenibacillus polymyxa* SC2 is positioned on the plasmid PPSC2_p0465. It is thus more likely that some, possibly even all, SpoIISA proteins from non-bacillus species were obtained through horizontal gene transfer, rather than from a common ancestor of bacillus and non-bacillus SpoIISA-bearing species. This is corroborated by the observation that the non-bacillus species do not form a single separate cluster in the phylogenetic tree, but are intermixed with sequences from bacillus species. Nevertheless, most SpoIISA proteins identified in this study come from species that are closely related to *B. cereus* or *B. subtilis* (Table S1), and the SpoIISA homologues can be grouped based on whether they originate from *B. subtilis* relatives or *B. cereus* relatives. SpoIISA similarity within each group is quite high (more than 90% for species from the *B. cereus* group and more than 80% for species from the more diverse *B. subtilis* group), but inter-group similarity is considerably lower (e.g., the similarity between the SpoIISA proteins from *B. cereus* E33L and from *B. subtilis* strain 168 is 45%, and the identity is only 20%). The similarity between the SpoIISA proteins from these two groups is thus comparable to the similarity between those located on chromosomes and those on plasmids (e.g., the similarity between *B. cereus* E33L and *Paenibacillus polymyxa* SC2 is 45.3%, and the identity is 19.6%).

Exactly one gene coding for SpoIISA was found in every available genome for strains from the *B. subtilis* and *B. cereus* groups. Moreover, the *spoIISA* gene was always found nearby well-conserved genes from the core genome, suggesting that in these species, *spoIISA* genes behave similarly as those from a very stable part of the core genome. There are two notable exceptions to this, however: *B. cereus* BDRD-ST196 possesses two SpoIISA proteins, one of which (NCBI-Protein ID: EEL06055) shows very high similarity (more than 95%) to SpoIISA proteins from other *B. cereus* strains and is positioned well inside the conserved core region of the genome, while the other one (NCBI-Protein ID: EEL06141) is only weakly similar to SpoIISA proteins from other *B. cereus* strains (about 30% identity and 45% similarity). The closest known relative to this second one is the SpoIISA protein (NCBI-Protein ID: WP_016427205) from *Paenisporosarcina* sp. HGH0030 (54% identity and 71% similarity). The second species with multiple SpoIISA proteins is *B. thuringiensis* Bt407, which contains three SpoIISA proteins. Again, one protein (NCBI-Protein ID: EEM28840) is very similar (more than 95%) to SpoIISA from closely-related strains and species, while the other two (NCBI-Protein ID: EEM25308 and EEM24943) differ considerably (less than 30% identity and less than 55% similarity to SpoIISA proteins from closely-related strains and species). The closest homologue of EEM25308 is again SpoIISA from *Paenisporosarcina* sp. HGH0030 (35% identity and 54% similarity), though they show only weak mutual similarity. The closest homologue of EEM24943 is SpoIISA from *B. simplex* (NCBI-Protein ID: CEG32548), but again, their mutual similarity is quite low (29% identity and 55% similarity). Interestingly, this protein is more similar to the SpoIISA proteins from the *B. subtilis* group (the identity to *B. amyloliquefaciens* LFB112 is 26%, similarity 50%) than to those from the *B. cereus* group (the identity to *B. cereus* E33L is 19%, similarity 46%) (Figure 1). Not only do EEM25308 and EEM24943 show low similarity to proteins from other species from the *B. cereus* group, but the proteins encoded by their neighboring genes are also dissimilar, making it probable that these genes are part of a flexible genome, which arose from horizontal gene transfer. It was recently reported that EEM24943 (locus: AFV22040; locus tag: BTB_502p07350) and EEM25308 (locus: AFV21663; locus tag: BTB_502p03580) are both positioned on plasmid BTB_502p [14]. The role that these multiple SpoIIS modules play in a cell is presently unknown, and many outstanding questions remain: Are all of these genes expressed or are the dissimilar ones nonfunctional pseudogenes? Are they all expressed during the same period in the cell cycle? In the same cell compartment? Do they interact with each other?

**Figure 2:**
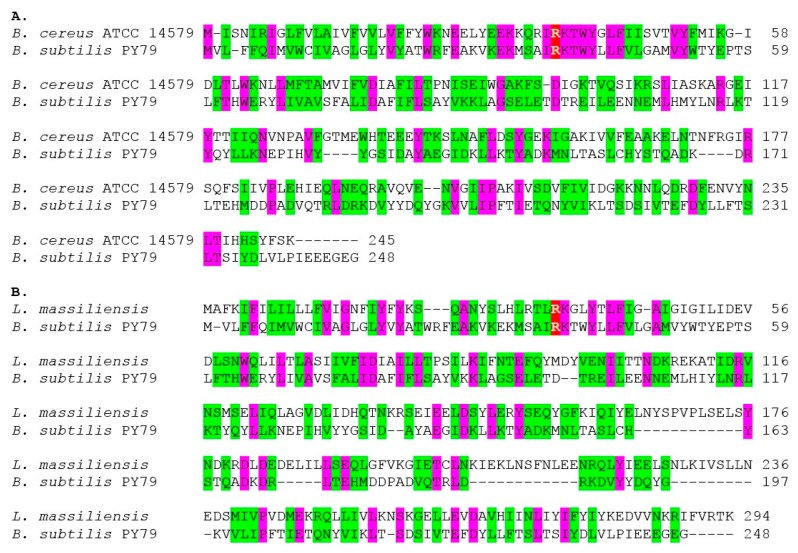
Comparison of two SpoIISA proteins. Amino acid sequence of the SpoIISA from *B. subtilis* PY79 is aligned with the SpoIISA from (**A**) *B. cereus* ATCC 14579 and (**B**) from *L. massiliensis*. Functionally-important residues are in white on a red background, identical residues are purple and similar ones are green.

An amino acid sequence comparison of the functionally-characterized SpoIISA proteins from *B. subtilis* PY79 (NCBI-Protein ID: AHA77284) and *B. cereus* ATCC 14579 (NCBI-Protein ID: AAP09399) shows that these two proteins contain 41 identical and 88 similar residues. The similarity between the SpoIISA proteins from *B. subtilis* PY79 and *Lysinibacillus massiliensis* (NCBI-Protein ID: KGR89544) is even higher (Figure 2), with 45 identical and 94 similar residues. Since a multiple sequence alignment of all of the SpoIISA proteins in our set showed that only a very few positions have identical or similar residues (Figure S1), it seems likely that the high level of similarity observed by comparing only these two SpoIISA proteins is not due to purifying selection, but is more likely a remnant of ancestral similarity. This assumes, of course, that all aligned sequences are from functional proteins. Since the presence of nonfunctional SpoIISA proteins could mask functionally-important positions, we aligned only those protein sequences containing the conserved, functionally-important arginine (Arg_38_ in *B. subtilis*) and the three *N*-terminal transmembrane helices, which were shown to be indispensable for SpoIISA functionality [5,15]. It is likely, however, that some essential features of SpoIISA are still undiscovered, and thus, we cannot be sure that all nonfunctional proteins were excluded from our final alignment. Comparison of the SpoIISA proteins from 56 different bacterial species revealed only four positions containing invariant residues. According to the *B. subtilis* numbering, they are Arg_38_, Tyr_42_, Asp_78_ and Asp_225_; eight positions possessed similar residues: Ile_37_, Lys_39_, Phe_82_, Lys_89_, Ile_143_, Leu_186_, Val_221_ and Leu_232_ (Figure S1). Most of these conserved residues seem to be located in the second and third transmembrane regions and in the *C*-terminal helix. Arg_38_, found near the beginning of the second transmembrane region, is important for oligomerization [15], and its substitution by glutamine results in loss of toxicity [5]. The function of the other conserved residues identified is presently unknown. The conserved aspartate (Asp_78_) in the third transmembrane region (Figure S1) may have an important functional role. It has been shown that an aspartate found in the transmembrane region of some receptors and transporters plays an important role in their operation [16,17,18,19]. Interestingly, a conserved aspartate (Asp_225_) is also present in the SpoIISA *C*-terminal α-helix. Some transmembrane prediction tools suggest that this helix is located in the membrane, but others do not [15]. Structural studies also failed to confirm the transmembrane localization of this helix, but its temporary presence in the membrane still cannot be ruled out [7]. The two conserved aspartates could play a role in SpoIISA assembly or multimerization, or they could be involved in some other process. Further functional and structural studies of the SpoIISA protein with substituted conserved aspartates could throw more light on their function.

### 2.2. Evolution of the SpoIISB and SpoIISC Antitoxins in Bacilli

**Figure 3:**
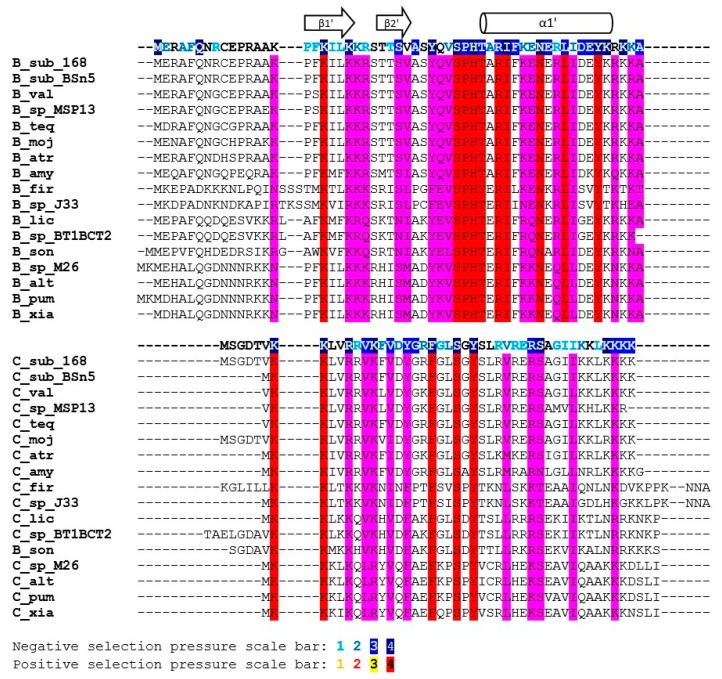
Multiple sequence alignment of the SpoIISB and SpoIISC amino acid sequences from the *B. subtilis* group. Residues that are identical within either the SpoIISB or SpoIISC proteins are red, and similar ones are purple. Selection pressure is indicated on the SpoIISB and SpoIISC reference sequences (from *B. subtilis* strain 168) shown above each sequence block. Selection pressure intensity is indicated by the given scale bar. SpoIISB secondary structure elements (as identified in [7]) are shown above the alignment; the abbreviations are from Table S3.

The sequence similarity of the SpoIISB and SpoIISC proteins is relatively high within the *B. subtilis* and *B. cereus* groups, but quickly decreases with growing evolutionary distance outside of these groups. Consequently, only SpoIISB and SpoIISC from the *B. subtilis* and *B. cereus* groups could be reliably identified and only sequences from these two groups were used in our study. We found SpoIISB proteins (hereafter SpoIISB_sub_) in all *B. subtilis* strains examined (Table S3), and the identity of SpoIISB_sub_ between all of these strains is more than 96%. The identity of SpoIISB_sub_ from *B. subtilis* to its homologues from closely-related species from the *B. subtilis* group (*B. vallismortis*, *B. tequilensis*, *B.* sp. MSP13, *B. mojavensis* and various *B. atrophaeus* strains) is also quite high, above 94%, with nearly all of the differences occurring in the first loop of the *N*-terminal part, which has been shown to be unnecessary for antitoxin activity [7]. Moving to more distantly-related species, SpoIISB_sub_ identity drops to 85% for various strains of *B. amyloliquefaciens* and is around 70%–75% for SpoIISB_sub_ from *B. pumilus*, *B. altitudinis*, *B. xiamenensis*, *B.* sp. DW5-4, *B.* sp. NSP9.1, *B. safensis*, *B.* sp. WP8, *B*. sp. BT1B_CT2, *B. sonorensis* and *B. licheniformis*. The *N*-terminal part again seems to harbor the substitutions. The shared, conserved regions could be identified by comparing the SpoIISB_sub_ proteins from the most distantly-related species (the identity of SpoIISB_sub_ from *B. subtilis* strain 168 to *B. firmus* and *B.* sp. J33 is 57%–59%). The most highly-conserved regions correspond approximately to the SpoIISB_sub_ secondary structure elements (Figure 3). Two of the most highly-conserved regions have been shown to be functionally important for antitoxin activity [7]. The first of these is β-strand β1′, whose three lysines are nearly invariant. This strand forms a two-stranded intermolecular antiparallel β-sheet with strand β1 of SpoIISA, and deletion of this part abolishes SpoIISB_sub_ antitoxin activity [7]. The second is a positively-charged *C*-terminal region comprising 2–4 tandem lysine or arginine residues (in *B. subtilis* K_52_RKK_55_). Although none of these four residues is invariant, they are always substituted with other hydrophilic residues (Figure 3). The interaction of this region with SpoIISA could not be inferred from the SpoIISAB complex structure, since it was not resolved, but its importance was clearly demonstrated by a deletion experiment: deletion of the last four *C*-terminal SpoIISB_sub_ residues obliterates the protein’s antitoxin activity [7]. Other conserved regions, whose function has not presently been identified, include β-strand β2′ and α-helix α1′ (the *N*-terminal edge of this helix is the most highly conserved region of the whole protein).

The identity of the SpoIISC proteins (hereafter SpoIISC_sub_) between the various *B. subtilis* strains is more than 93%. The identity of *B. subtilis* SpoIISC_sub_ to its homologues from closely-related species (*B. vallismortis*, *B. tequilensis*, *B.* sp. MSP13, *B. mojavensis*) is comparable, from 90%–97%. The identity to its homologues from various *B. atrophaeus* strains, which is also a close relative, is lower, around 90%, but almost all substitutions are for residues that are functionally similar (96% positives). Moving to more distant relatives, the *B. subtilis*/*B. amyloliquefaciens* identity is 87%, with most substitutions concentrated in the *C*-terminal half of the protein, the region corresponding to the middle part of the α1′-helix in SpoIISB_sub_. The identity drops to 75% for SpoIISC_sub_ homologues from *B. licheniformis* WX-02 and *B.* sp. CPSM8, and the substitutions are now distributed equally throughout the protein (Figure 3). The identity of *B. subtilis* SpoIISC_sub_ to its homologues from the more distantly-related members of the *B. subtilis* group (*B. pumilus*, *B.* sp. M 2–6, *B*. *altitudinis* 41KF2b, *B. xiamenensis*) is 68%, and the identity to the most distant SpoIISC_sub_ homologs (from *B. firmus* and *B.* sp. J33) is 55%–60%. Since the identities of the SpoIISC_sub_ proteins from various species of the *B. subtilis* group do not differ much from the identities observed for the SpoIISB_sub_ proteins, it seems likely that both the SpoIISB_sub_ and SpoIISC_sub_ proteins from all of these organisms were inherited vertically and not obtained through horizontal gene transfer. In contrast to SpoIISB_sub_, only a few SpoIISC_sub_ residues are invariant. Two conserved lysines are present at the *N*-terminus (K_2_K_3_ in *B. subtilis* BSn5), and a conserved serine and tyrosine appear in the central part of the protein (S_19_XY_21_). A *C*-terminal stretch of 3–4 positively-charged residues (Lys or Arg) is also well conserved (Figure 3).

SpoIISB_sub_ proteins show only limited similarity to SpoIISC_sub_ proteins. For example, the SpoIISB_sub_ and SpoIISC_sub_ proteins from *B. subtilis* strain 168 are only 14.3% identical (39.3% similar). They also differ in length: SpoIISB_sub_ from the *B. subtilis* 168 strain is 56 residues long, while its SpoIISC_sub_ is only 45 residues. Since the exact position of the start and stop codons was not identified, however, the real proteins could be slightly longer or shorter than this. SpoIISB_sub_ and SpoIISC_sub_ do share two stretches of positively-charged residues near their *C*- and *N*-terminal ends (according to *B. subtilis* strain 168 numbering, the conserved, shared residues are K_15_XXKIL(LV)KK_22_ at the *N*-terminus and K_52_R(K)KK_55_ at the *C*-terminus). The central parts of these proteins are also well conserved (Y_31_XV(L)**S**XH(Y)T(S)X**R**I(V)_40_), with two invariant residues, a serine (Ser_34_) and an arginine (Arg_39_) (Figure 3). However, comparing SpoIISB_sub_ and SpoIISC_sub_ from multiple species reveals that only the first three of the four *N*-terminal positively-charged residues are conserved (according to *B. subtilis* strain 168 numbering K_15_X_var_KXXK). Even more strikingly, only one of the conserved positively-charged residues at the *C*-terminus (K_53_) is completely conserved across all proteins (Figure 3). As noted, deletion of the last four SpoIISB_sub_ residues considerably decreased sporulation efficiency. It is thus possible that it was the deletion of just this universally-conserved residue that was responsible for this effect. Measuring the sporulation efficiency of a point mutant in this lysine would be needed to corroborate this hypothesis, however. Of the two remaining residues common to both SpoIISB_sub_ and SpoIISC_sub_, Ser_34_ was conserved in all studied SpoIISB_sub_ and SpoIISC_sub_ proteins, but Arg_39_ was not; the significance of this is presently unclear. Finally, for whatever reason, conservation of the SpoIISC_sub_ proteins is more relaxed than the conservation of the SpoIISB_sub_ proteins. This is most clearly seen by comparing the SpoIISB_sub_ and SpoIISC_sub_ proteins from *B. subtilis* and *B. pumilus*. The similarity between the SpoIISB_sub_ proteins from the two species is 81.4% (65.1% identity), and the similarity between the SpoIISC_sub_ proteins is only 58.1% (32.6% identity).

**Figure 4:**
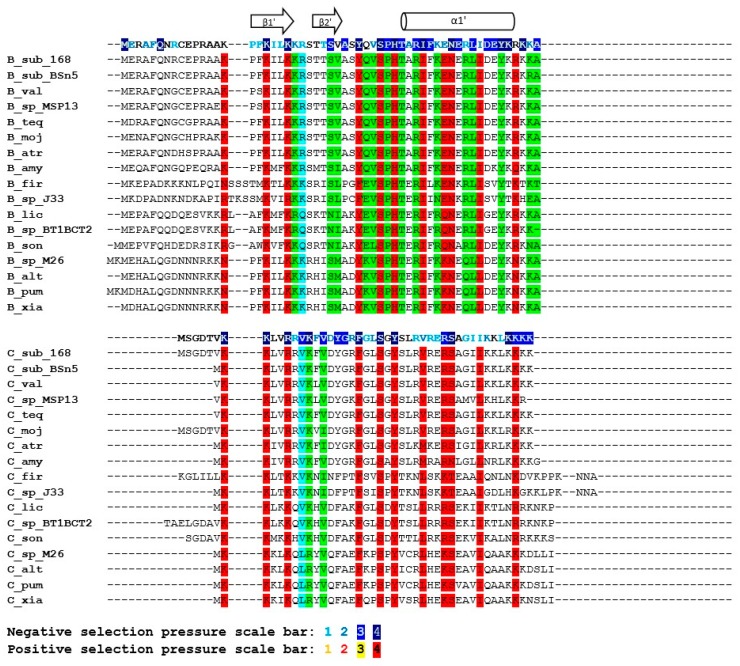
Conserved positions in the SpoIISB and SpoIISC proteins from the *B. subtilis* group. Positions containing identical or similar residues in both groups are colored red; positions containing identical or similar residues in one, but not the other group are green. Positions conserved in both groups, but containing dissimilar residues, are cyan. Selection pressure intensity and secondary structure elements are indicated as in Figure 3. The abbreviations are from Table S3.

Since *spoIISB_sub_* and *spoIISC_sub_* are positioned adjacently on the chromosome, exhibiting sequence similarity, it is likely that they are the result of a gene duplication event. Further evidence for this comes from the recent report that both proteins have similar functions [8]: they are both able to interact with and neutralize the toxicity of SpoIISA. An earlier report [5] showed that a sporulation defect was still observed when only *spoIISB_sub_* (and not *spoIISC_sub_*) was inactivated [5]. The most likely reason for the earlier observation was that the insertion of the tetracycline resistance cassette into codon 17 of *spoIISB*_sub_ might have disrupted a potential *spoIISC*_sub_ promoter positioned inside the *spoIISB*_sub_ gene. The SpoIISC protein also did not compensate for *mut*14, which deactivated *spoIISB_sub_*, but *mut*14 is a frameshift mutation caused by the deletion of 2 bp after codon 52 of *spoIISB*_sub_ [5], and this mutation would also be expected to affect a *spoIISC*_sub_ promotor or regulatory region. Alternatively, *spoIISC_sub_* might have been deactivated by its own, independent mutation, a possibility that was not explored in the original report [5]. A final possibility is that SpoIISC_sub_ is expressed at a different stage of the cell cycle and therefore would not have been available to compensate for the loss of SpoIISB_sub_. As noted earlier, different gene expression profiles could be one reason why there are two *spoIIS* antitoxin genes present. Generally, after gene duplication, one of the copies becomes redundant and usually goes through a pseudogenization process in which it neutrally accepts nonsynonymous or even deleterious mutations and subsequently becomes nonfunctional or is lost. It may also obtain a new function, different from that of the other copy or the copies may also undergo a division of labor, with each copy retaining a different sub-function of the original, ancestral function. We thus searched for differences between the amino acid sequences of SpoIISB_sub_ and SpoIISC_sub_, which could indicate either a division of labor or the gain of a new function. We found one position, conserved in both proteins, that contains dissimilar residues. A polar residue (arginine, lysine or glutamine; Arg_23_ in *B. subtilis*) in SpoIISB_sub_ is replaced by a nonpolar residue (valine or leucine) in SpoIISC_sub._ The implication of this change remains to be investigated, but it could make SpoIISC_sub_ bind less (Figure 4). The alignment in Figure 4 shows that the SpoIISB_sub_ proteins contain more (twelve) conserved, non-shared positions than SpoIISC_sub_ (only two). Since these proteins have no reported function other than inhibiting SpoIISA, it is difficult to imagine how an ancestral function could be subdivided between them. It is possible (although it has not yet been investigated) that they differ in binding strength to SpoIISA and that this difference could explain why SpoIISB_sub_ contains more conserved positions than SpoIISC_sub_. For example, SpoIISB_sub_ might contain more conserved positions than SpoIISC_sub_ because more of its residues are involved in SpoIISA interactions, resulting in stronger binding. It is not clear what advantage the presence of two inhibitors of different binding strengths would give to a cell, suggesting that SpoIISC_sub_ might have an additional function. The most likely possibility is that SpoIISC_sub_ (or SpoIISB_sub_) when bound to SpoIISA can regulate the expression of the *spoIIS* operon, making it serve as the third regulatory component of a three-component TA system [20,21,22]. However, the SpoIIS cluster, with its two antitoxin proteins, would differ from other known three-component systems, which are formed by a toxin, an antitoxin and a regulatory protein, in that no regulatory proteins have yet been found that also exhibit antitoxin capabilities. On the contrary, it has been shown that PasC, the third component of the plasmid addiction system from *Thiobacillus ferrooxidans* plasmid pTF-FC2, is not capable of neutralizing the toxicity of the PasB toxin on its own [20].

**Figure 5:**
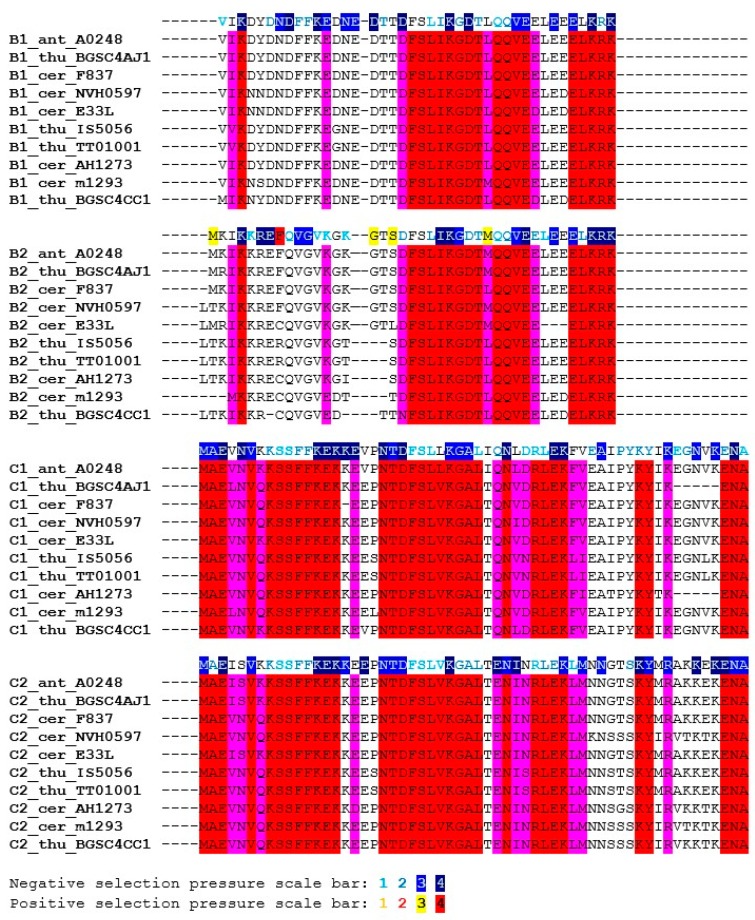
Multiple sequence alignment of the SpoIISB and SpoIISC amino acid sequences from the *B. cereus* group. Residues that are identical in either SpoIISB or SpoIISC are red, and similar ones are purple. Selection pressure is indicated on the SpoIISB and SpoIISC reference sequences (from *B. anthracis* A0248) shown above each sequence block. Selection pressure intensity is indicated by the given scale bar. The abbreviations are from Table S3.

**Figure 6:**
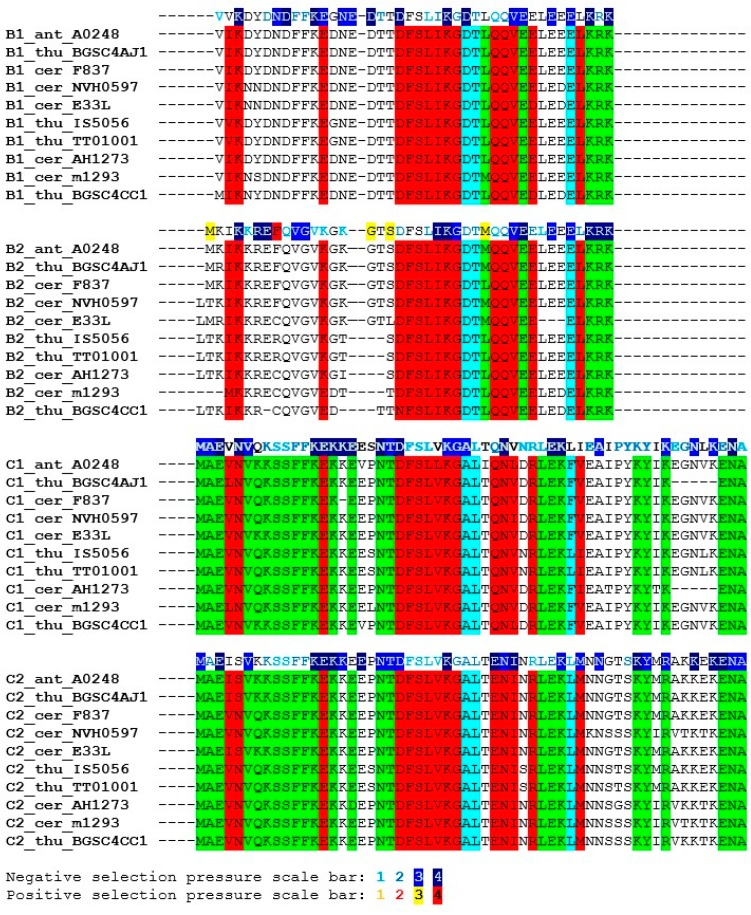
Conserved positions in the SpoIISB and SpoIISC proteins from the *B. cereus* group. Positions in both groups containing identical or similar residues are colored red; positions containing identical or similar residues in one, but not the other group are green. Positions conserved in both groups, but containing dissimilar residues are cyan. Selection pressure intensity is indicated as in Figure 5. The abbreviations are from Table S3.

SpoIISB_cer_ proteins are present in all members of the *B. cereus* group. Some *B. cereus* strains possess two SpoIISB_cer_ (SpoIISB1_cer_, SpoIISB2_cer_,) and two SpoIISC_cer_ (SpoIISC1_cer_, SpoIISC2_cer_) proteins. The differences between them will be explored in Section 2.3 below. The identity between SpoIISB_cer_ from various *B. cereus* strains and from closely-related species like *B.* *anthracis* and *B. thuringiensis* is more than 80% (similarity is more than 90%). The identity between SpoIISB_cer_ proteins from more distantly-related members of the *B. cereus* group (e.g., between *B. cereus* E33L and *B. mycoides* Rock1-4 SpoIIB_cer_) is more than 70% (the similarity is more than 85%). The most conserved part is an eight-residue central region, including residues D_19_FSLIKGD_26_ (*B. cereus* m1293 numbering) and the *C*-terminal region. The *N*-terminal region, except for the highly-conserved K_3_ and a F_9_FK_11_ motif, is less conserved (Figure 5). Similarly, SpoIISC_cer_ proteins are present in all members of the *B. cereus* group. The identity between SpoIISC_cer_ from various *B. cereus* strains and from closely-related species like *B.* *anthracis* and *B. thuringiensis* is more than 80% (similarity is more than 90%). The identity between SpoIISC_cer_ proteins from more distantly-related members of the *B. cereus* group (e.g., between *B. cereus* E33L and *B. mycoides* Rock1-4 SpoIISC_cer_) is more than 60% (the similarity is more than 80%). The SpoIISC_cer_ proteins are all about 16 residues longer than their corresponding SpoIISB_cer_ proteins (e.g., *B. cereus* m1293 SpoIISB_cer_ is 41 residues long, while its SpoIISC_cer_ is 58 residues). The conservation pattern in SpoIISC_cer_ is very similar to that of SpoIISB_cer_: the lower similarity at the *N*-terminus and the higher similarity at the *C*-terminus. Both SpoIISB_cer_ and SpoIISC_cer_ proteins have an *N*-terminal polar residue (K_3_ in *B. cereus* m1293), an FFK motif (F_9_FK_11_), a DFSLI(V)KG motif (D_19_FSLIKG_25_) and a conserved *C*-terminal region. The SpoIISC_cer_ proteins contain an additional 12 residues at the *C*-terminus (Figure 5), but this region is not especially well conserved. Like their *B. subtilis* counterparts, the SpoIISC_cer_ proteins are less tightly conserved than the corresponding SpoIISB_cer_ proteins. For example, the similarity between the SpoIISB_cer_ proteins from *B. cereus* m1293 and *B. cereus* AH620 is 97.6% (87.8% identity), while the similarity between the corresponding SpoIISC_cer_ proteins is 89.7% (84.5% identity). Since the genes coding for SpoIISB_cer_ and SpoIISC_cer_ proteins are positioned next to each other on the chromosome and show a high degree of sequence similarity, it is likely that they are the result of a gene duplication event. A search for signs of either division of labor or gain of function yielded three interesting positions. In SpoIISB_cer_, a conserved aspartate (Asp_26_ in *B. cereus* m1293) appears after the DFSLI(V)KG motif, but in SpoIISC_cer_, an alanine is found instead (Figure 6). The following residue in SpoIISB_cer_, a conserved threonine (Thr_27_ in *B. cereus* m1293), is replaced by leucine in SpoIISC_cer_. Finally, a negatively-charged glutamate appears 10 positions downstream of this threonine in SpoIISB_cer_ (Glu_37_ in *B. cereus* m1293), but is replaced by the aromatic phenylalanine (or the aliphatic leucine) in SpoIISC_cer_ (Figure 6). We also examined the selection pressure acting upon the SpoIISB_cer_ and SpoIISC_cer_ proteins, but no significant differences could be inferred (Figure 5 and Figure 6). Only a weak negative selection signal was detected in the highly-conserved central region. The extremely high level of conservation in this region, which is almost invariant even at the DNA level, provides only a low level of statistical support for negative selection, which is the most likely reason for this weak signal.

### 2.3. Evolution of the SpoIIS Module

It might be speculated that SpoIISA acts as a holin, forming a pore in the cytoplasmic membrane through which some lytic protein, e.g., an amidase, is released to lyse the cell wall peptidoglycan. Intriguingly, the *B. subtilis* *spoIIS* cluster partially overlaps the *N*-acetylmuramoyl-l-alanine amidase (XlyA) of prophage PBSX (although in the opposite direction). Inspection of the gene arrangement in various species reveals, however, that the *spoIIS* cluster is positioned near the PBSX prophage only in species from the *B. subtilis* group and that this connection is missing in the more distantly-related *spoIIS* cluster-bearing species. Although an evolutionary relationship, or some past or present interaction, between the *spoIIS* cluster and the PBSX prophage cannot be ruled out, expression of the *spoIIS* cluster from *B. subtilis* and *B. cereus* in *E. coli* cells shows that SpoIISA is able to induce cell lysis without the help of XlyA or any other prophage protein [6].

**Figure 7:**
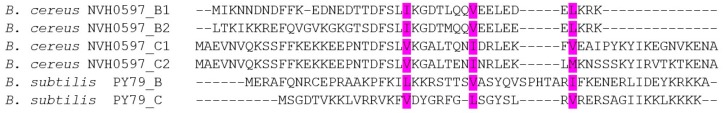
Multiple sequence alignment of SpoIISB and SpoIISC amino acid sequences from two representatives from the *B. cereus* and the *B. subtilis* groups. Similar residues are colored purple.

**Figure 8:**
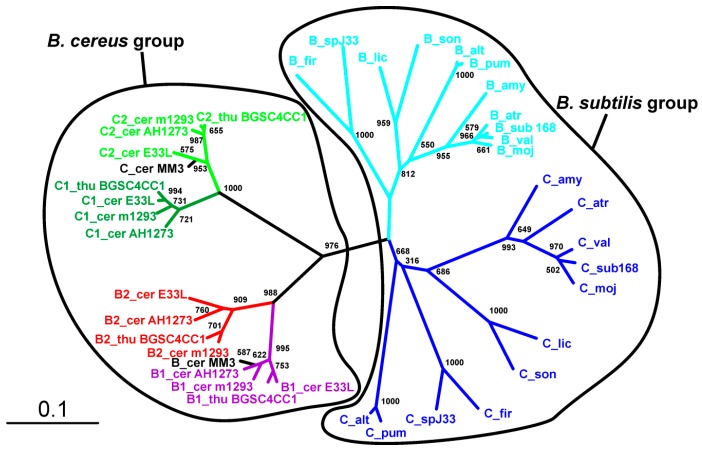
Phylogeny of the SpoIISB and SpoIISC proteins. Neighbor-joining phylogenetic tree based on the SpoIISB and SpoIISC protein sequences from selected species. The SpoIISB_sub_ proteins are colored cyan; the SpoIISC_sub_ proteins are blue; the SpoIISB1_cer_ proteins are magenta; the SpoIISB2_cer_ proteins are red; the SpoIISC1_cer_ proteins are dark green; the SpoIISC2_cer_ proteins are light green; and the SpoIISB_cer_ and SpoIISC_cer_ proteins from the short form are black. The scale bar shows evolutionary distance in the number of amino acid substitutions per site. The abbreviations are from Table S3.

**Figure 9:**
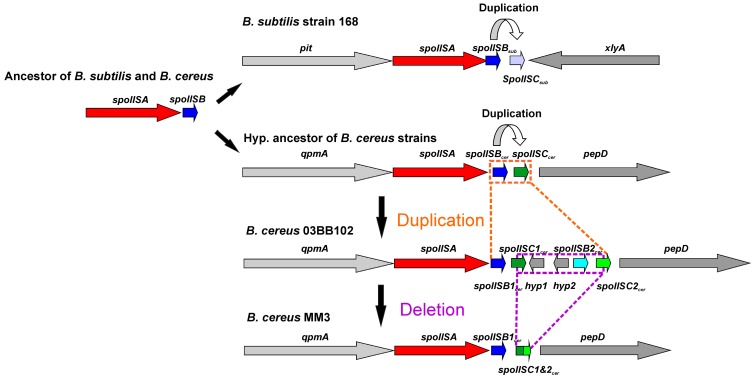
Possible evolution of the SpoIIS module in the *B. cereus* group. Two SpoIIS module architecture types are found in the *B. cereus* group. The shorter of the two consists of the *spoIISA* gene followed by the *spoIISB_cer_* and *spoIISC_cer_* genes; the longer one comprises the *spoIISA* gene followed by the *spoIISB1_cer_* and *spoIISC1_cer_* genes, then two genes for unknown hypothetical proteins and, finally, the *spoIISB2_cer_* and *spoIISC2_cer_* genes. The shorter form evolved from the longer one by deletion of the middle part. The evolutionary scenario shown assumes a cleavage site positioned inside the *spoIISC1_cer_* gene.

The overall similarity between SpoIISB and SpoIISC from species of *B. cereus* and *B. subtilis* groups is very low, with no invariant residues and only three positions containing relatively similar residues (Figure 7). Thus, they either shared a common ancestor and afterwards evolved beyond recognition or they originated independently. Common ancestry seems the more probable explanation, since it is hard to imagine that so potent of a toxin as SpoIISA could exist in a viable cell without an inhibitor. A given SpoIISB or SpoIISC protein from a species belonging to one of the two groups is more similar to another SpoIISB or SpoIISC (either one) from the same group than it is to its corresponding orthologue from a species from the second group. This can be most clearly seen in the phylogenetic tree of SpoIISB and SpoIISC (Figure 8) where the SpoIIB_sub_ and SpoIISC_sub_ proteins from the *B. subtilis* group are positioned on a common branch, which is isolated from the branch occupied by the SpoIISB_cer_ and SpoIISC_cer_ proteins from the *B. cereus* group. It thus seems very likely that the SpoIISB and SpoIISC proteins are the result of two independent gene duplication events, which took place after the *B. cereus* and *B. subtilis* groups separated (Figure 9, upper part). Although the SpoIISB and SpoIISC proteins in a given organism are clearly paralogues, because the gene duplication events appear to have happened after the *B. subtilis* and *B. cereus* groups separated, the SpoIISB and SpoIISC proteins from species belonging to different groups are not clearly orthologues. Interestingly, species (and strains) from the *B. cereus* group have two different architectures for the SpoIIS module. There is a shorter one, consisting of the *spoIISA* gene followed by the *spoIISB*_cer_ and *spoIISC*_cer_ genes, and a longer one comprising the *spoIISA* gene followed by the *spoIISB1_cer_* and *spoIISC1_cer_* genes, then two genes for unknown hypothetical proteins and, finally, the *spoIISB2_cer_* and *spoIISC2_cer_* genes. Although one might expect that the longer form is the result of duplicating the shorter form, the opposite seems to be true. As the phylogenetic tree (Figure 8) shows, SpoIISB_cer_ from the short form corresponds to SpoIISB1_cer_ from the long form, while SpoIISC_cer_ from the short form corresponds to SpoIISC2_cer_ (or at least the *C*-terminal part of it) from the long form. The most parsimonious explanation for these observations is that the middle part of the longer form was deleted (Figure 9, lower part). Since the intergenic regions between *spoIISB1_cer_* and *spoIISC1_cer_* and *spoIISB2_cer_* and *spoIISC2_cer_*, as well as the *N*-terminal parts of *spoIISC1_cer_* and *spoIISC2_cer_* are identical in the longer form, it is difficult to determine the exact position of the cleavage site. If the cleavage site was positioned inside the intergenic region, the deleted part would comprise the entirety of the *spoIISC1_cer_* and *spoIISB2_cer_* genes, leaving the complete sequences of the *spoIISB1_cer_* and *spoIISC2_cer_* genes in the short form. Alternatively, if the cleavage site was positioned inside *spoIISC1_cer_*, then the *C*-terminal part of *spoIISC1_cer_*, all of *spoIISB2_cer_* and the *N*-terminal part of *spoIISC2_cer_* would be deleted. *SpoIISB_cer_* of the short form would then correspond to *spoIISB1_cer_*, and *spoIISC_cer_* would have been formed by the fusion of the *C*-terminal part of *spoIISC1_cer_* and the *N*-terminal part of *spoIISC2_cer_* from the longer form (Figure 9). Notably, we found signs of nonfunctionalization in the *spoIISC1_cer_* and *spoIISB1_cer_* genes in some strains of *B. cereus*, including deletions, nonsense mutations and reading-frame shifts. It is thus possible that these two genes were either pseudogenes (or were on their way to becoming ones) even before they were deleted. The high mutual sequence similarity between the *spoIISB1_cer_*/*spoIISC1_cer_* and *spoIISB2_cer_*/*spoIISC2_cer_* pairs and the presence of pseudogenes in the middle part of this region thus poised it for deletion.

## 3. Conclusions

In most species of the Bacillus genus of the Firmicutes phylum, we found exactly one copy of the *spoIISA* gene, which was positioned next to well-conserved core genes, indicating that they behave as the stable part of the core genome. We also found another group of SpoIISA proteins in non-bacillus species, which were probably obtained from Bacillus species through horizontal gene transfer. Comparing the SpoIISA proteins from 56 different bacterial species revealed that only four positions carried invariant residues and eight positions possessed similar residues (with the caveat that some important residues might have been masked by the possible presence of non-functional proteins in our dataset). Interestingly, one of these conserved residues is an aspartate located in the third transmembrane region. All Bacillus species studied possess two adjacent antitoxin genes, which are likely the result of gene duplication. This gene duplication occurred independently in the *B. subtilis* and *B. cereus* lineages after they had separated. Although the reason for the presence of two antitoxin genes still needs to be investigated, it is clear that the SpoIISB protein contains more conserved residues than the SpoIISC protein, whose conservation seems to be more relaxed. In the *B. cereus* lineage, the *spoIIS* module is present in two different architectures. The shorter of the two consists of the *spoIISA_cer_* gene followed by the *spoIISB_cer_* and *spoIISC_cer_* genes, while the longer one comprises the *spoIISA* gene followed by the *spoIISB1_cer_* and *spoIISC1_cer_* genes, then genes for two unknown hypothetical proteins and, finally, *spoIISB2_cer_* and *spoIISC2_cer_*. A phylogenetic analysis suggests that the short form evolved from the long form by deletion of the middle part.

## 4. Experimental Section

The amino acid sequences of the SpoIISA, SpoIISB and SpoIISC proteins from various bacterial species were obtained using protein BLAST [23] against the non-redundant database. Amino acid sequences of the characterized SpoIISA (NCBI-Protein ID: AHA77284), SpoIISB_sub_ (NCBI-Protein ID: AHA77283) [5,6] and SpoIISC_sub_ (NCBI-Protein ID: AHA77282) proteins from *B. subtilis* PY79 and the SpoIISA (NCBI-Protein ID: AAP09399) and SpoIISC_cer_ (NCBI-Protein ID: AAP09400) proteins from *B. cereus* ATCC 14579 [8] were used as queries. Since the amino acid sequence of SpoIISB_cer_ from *B. cereus* ATCC 14579 is not found in the NCBI protein database, it was translated directly from the genomic sequence [24] and then used as the BLAST search query. If the amino acid sequences of the SpoIIS proteins from a given bacterial species were not found using BLAST, they were produced by translating from the appropriate genomic sequence. The resulting amino acid sequences were then aligned using ClustalX [25]. Nucleic acid sequences of particular *spoIIS* genes were obtained from the bacterial genomes available in GenBank [26]. A neighbor-joining (NJ) tree [27] was calculated using ClustalX using default parameters, and the tree topology reliability was evaluated using the bootstrap test [28] with 1000 replications. Selection pressure was estimated using the SELECTON tool [29] using the M8 codon-substitution model [30] and a neighbor-joining tree. For calculating selection pressure, the sequences listed in Table S3 were used. Sequence similarities were estimated using MatGAT [31] using the BLOSUM62 matrix.

## Supplementary Materials

The following are available online at www.mdpi.com/2072-6651/8/6/180/s1. Figure S1: Multiple sequence alignment of SpoIISA amino acid sequences from various bacterial species. Functionally-important residues are white on a red background; identical residues are purple; similar ones are green. Substitutions of functionally-important residues with dissimilar residues are colored cyan. Secondary structure elements (as identified in [7]) are shown above the alignment. Table S1: The SpoIISA proteins from Bacillus and non-Bacillus species and species with available genome sequences lacking the SpoIISA proteins. Table S2: The SpoIISA proteins used in phylogenetic analysis. Table S3: SpoIISB and SpoIISC proteins from the species of the *B. subtilis* and *B. cereus* groups.

## References

[1] Engelberg-Kulka H., Amitai S., Kolodkin-Gal I., Hazan R. (2006). Bacterial programmed cell Death and multicellular behavior in bacteria. PLoS Genet..

[2] Page R., Peti W. (2016). Toxin-antitoxin systems in bacterial growth arrest and persistence. Nat. Chem. Biol..

[3] Goeders N., van Melderen L. (2014). Toxin-antitoxin systems as multilevel interaction systems. Toxins.

[4] Aakre C.D., Phung T.N., Huang D., Laub M.T. (2013). A bacterial toxin inhibits DNA replication elongation through a direct interaction with the β sliding clamp. Mol. Cell.

[5] Adler E., Barák I., Stragier P. (2001). *Bacillus subtilis* locus encoding a killer protein and its antidote. J. Bacteriol..

[6] Florek P., Muchová K., Pavelčíková P., Barák I. (2008). Expression of functional Bacillus SpoIISAB toxin-antitoxin modules in *Escherichia coli*. FEMS Microbiol. Lett..

[7] Florek P., Levdikov V.M., Blagova E., Lebedev A.A., Škrabana R., Rešetárová S., Pavelčíková P., Barak I., Wilkinson A.J. (2011). The structure and interactions of SpoIISA and SpoIISB, a toxin-antitoxin system in *Bacillus subtilis*. J. Biol. Chem..

[8] Melničáková J., Bečárová Z., Makroczyová J., Barák I. (2015). Analysis of the *Bacillus cereus* SpoIIS antitoxin-toxin system reveals its three-component nature. Front. Microbiol..

[9] Eppinger M., Bunk B., Johns M.A., Edirisinghe J.N., Kutumbaka K.K., Koenig S.S., Creasy H.H., Rosovitz M.J., Riley D.R., Daugherty S. (2011). Genome sequences of the biotechnologically important *Bacillus megaterium* strains QM B1551 and DSM319. J. Bacteriol..

[10] Liu L., Li Y., Zhang J., Zou W., Zhou Z., Liu J., Li X., Wang L., Chen J. (2011). Complete genome sequence of the industrial strain *Bacillus megaterium* WSH-002. J. Bacteriol..

[11] Switzer Blum J., Burns Bindi A., Buzzelli J., Stolz J.F., Oremland R.S. (1998). *Bacillus arsenicoselenatis*, sp. nov., and *Bacillus selenitireducens*, sp. nov.: two haloalkaliphiles from Mono Lake, California that respire oxyanions of selenium and arsenic. Arch. Microbiol..

[12] Takami H., Nakasone K., Takaki Y., Maeno G., Sasaki R., Masui N., Fuji F., Hirama C., Nakamura Y., Ogasawara N. (2000). Complete genome sequence of the alkaliphilic bacterium *Bacillus halodurans* and genomic sequence comparison with *Bacillus subtilis*. Nucleic Acids Res..

[13] Janto B., Ahmed A., Ito M., Liu J., Hicks D.B., Pagni S., Fackelmayer O.J., Smith T.A., Earl J., Elbourne L.D. (2011). Genome of alkaliphilic *Bacillus pseudofirmus* OF4 reveals adaptations that support the ability to grow in an external pH range from 7.5 to 11.4. Environ. Microbiol..

[14] Sheppard A.E., Poehlein A., Rosenstiel P., Liesegang H., Schulenburg H. (2013). Complete Genome Sequence of *Bacillus thuringiensis* Strain 407 Cry-. Genome Announc..

[15] Makroczyová J., Rešetárová S., Florek P., Barák I. (2014). Topology of the *Bacillus subtilis* SpoIISA protein and its role in toxin-antitoxin function. FEMS Microbiol. Lett..

[16] Kong H., Raynor K., Yasuda K., Moe S.T., Portoghese P.S., Bell G.I., Reisine T. (1993). A single residue, aspartic acid 95, in the delta opioid receptor specifies selective high affinity agonist binding. J. Biol. Chem..

[17] Befort K., Tabbara L., Bausch S., Chavkin C., Evans C., Kieffer B. (1996). The conserved aspartate residue in the third putative transmembrane domain of the delta-opioid receptor is not the anionic counterpart for cationic opiate binding but is a constituent of the receptor binding site. Mol. Pharmacol..

[18] Cavalli A., Babey A.M., Loh H.H. (1999). Altered adenylyl cyclase responsiveness subsequent to point mutations of Asp 128 in the third transmembrane domain of the delta-opioid receptor. Neuroscience.

[19] Jindrichova M., Vavra V., Obsil T., Stojilkovic S.S., Zemkova H. (2009). Functional relevance of aromatic residues in the first transmembrane domain of P2X receptors. J. Neurochem..

[20] Smith A.S., Rawlings D.E. (1997). The poison-antidote stability system of the broad-host-range *Thiobacillus ferrooxidans* plasmid pTF-FC2. Mol. Microbiol..

[21] Zielenkiewicz U., Ceglowski P. (2005). The toxin-antitoxin system of the streptococcal plasmid pSM19035. J. Bacteriol..

[22] Hallez R., Geeraerts D., Sterckx Y., Mine N., Loris R., Van Melderen L. (2010). New toxins homologous to ParE belonging to three-component toxin-antitoxin systems in *Escherichia coli* O157:H7. Mol. Microbiol..

[23] Altschul S.F., Gish W., Miller W., Myers E.W., Lipman D.J. (1990). Basic local alignment search tool. J. Mol. Biol..

[24] Ivanova N., Sorokin A., Anderson I., Galleron N., Candelon B., Kapatral V., Bhattacharyya A., Reznik G., Mikhailova N., Lapidus A. (2003). Genome sequence of *Bacillus cereus* and comparative analysis with *Bacillus anthracis*. Nature.

[25] Larkin M.A., Blackshields G., Brown N.P., Chenna R., McGettigan P.A., McWilliam H., Valentin F., Wallace I.M., Wilm A., Lopez R. (2007). Clustal W and Clustal X version 2.0. Bioinformatics.

[26] Benson D.A., Cavanaugh M., Clark K., Karsch-Mizrachi I., Lipman D.J., Ostell J., Sayers E.W. (2013). GenBank. Nucleic Acids Res..

[27] Saitou N., Nei M. (1987). The neighbor-joining method: A new method for reconstructing phylogenetic trees. Mol. Biol. Evol..

[28] Felsenstein J. (1981). Evolutionary trees from DNA sequences: A maximum likelihood approach. J. Mol. Evol..

[29] Doron-Faigenboim A., Stern A., Mayrose I., Bacharach E., Pupko T. (2005). Selecton: A server for detecting evolutionary forces at a single amino-acid site. Bioinformatics.

[30] Yang Z., Nielsen R., Goldman N., Pedersen A.M. (2000). Codon-substitution models for heterogeneous selection pressure at amino acid sites. Genetics.

[31] Campanella J.J., Bitincka L., Smalley J. (2003). MatGAT: An application that generates similarity/identity matrices using protein or DNA sequences. BMC Bioinform..

